# Protocol for the *S*moking, *N*icotine *a*nd *P*regnancy (SNAP) trial: double-blind, placebo-randomised, controlled trial of nicotine replacement therapy in pregnancy

**DOI:** 10.1186/1472-6963-7-2

**Published:** 2007-01-03

**Authors:** Tim Coleman, Jim Thornton, John Britton, Sarah Lewis, Kim Watts, Michael WH Coughtrie, Clare Mannion, Neil Marlow, Christine Godfrey

**Affiliations:** 1 Division of Primary Care, University of Nottingham, Nottingham, UK; 2 Division of Obstetrics and Gynaecology, University of Nottingham, Nottingham, UK; 3 Division of Respiratory Medicine, University of Nottingham, Nottingham, UK; 4 Academic Division of Midwifery, University of Nottingham, Nottingham, UK; 5 Department of Molecular & Cellular Pathology, University of Dundee, Dundee, UK; 6 Stop Smoking Service, Central Cheshire PCT, Crewe, UK; 7 Academic Division of Child Health, University of Nottingham, Nottingham, UK; 8 Department of Health Sciences, University of York, York, UK

## Abstract

**Background:**

Smoking in pregnancy remains a public health challenge. Nicotine replacement therapy (NRT) is effective for smoking cessation in non-pregnant people, but because women metabolise nicotine and cotinine much faster in pregnancy, it is unclear whether this will be effective for smoking cessation in pregnancy. The NHS Health Technology Assessment Programme (HTA)-funded smoking, nicotine and pregnancy (SNAP) trial will investigate whether or not nicotine replacement therapy (NRT) is effective, cost-effective and safe when used for smoking cessation by pregnant women.

**Methods/Design:**

Over two years, in 5 trial centres, 1050 pregnant women who are between 12 and 24 weeks pregnant will be randomised as they attend hospital for ante-natal ultrasound scans. Women will receive either nicotine or placebo transdermal patches with behavioural support. The primary outcome measure is biochemically-validated, self-reported, prolonged and total abstinence from smoking between a quit date (defined before randomisation and set within two weeks of this) and delivery. At six months after childbirth self-reported maternal smoking status will be ascertained and two years after childbirth, self-reported maternal smoking status and the behaviour, cognitive development and respiratory symptoms of children born in the trial will be compared in both groups.

**Discussion:**

This trial is designed to ascertain whether or not standard doses of NRT (as transdermal patches) are effective and safe when used for smoking cessation during pregnancy.

## Background

Maternal smoking during pregnancy harms unborn children and, as up to 30% of pregnant women smoke [1], it is a significant public health problem. The adverse effects of smoking during pregnancy include an increased risk of miscarriage and stillbirth, accounting for 4000 deaths annually, and of pre-term birth and low birth weight leading to increased perinatal morbidity [2,3]. Children of mothers who smoke whilst pregnant are at increased risk of neo-natal mortality, sudden infant death syndrome and asthma [2]. Maternal smoking whilst pregnant is also associated with an increased risk of attention deficit and learning problems in childhood [3,4]. Currently only around 25% of pregnant smokers stop for even part of their pregnancy and, of these, around two thirds re-start post-natally [1].

Effective methods for promoting smoking cessation by pregnant women are required. The most effective smoking cessation therapy in non-pregnant smokers is a combination of behavioural support and pharmacotherapy with either nicotine replacement therapy (NRT) [5] or bupropion [6]. Behavioural support alone can increase smoking cessation rates by up to 7% [7] and the addition of pharmacotherapy increases this further by 1.5 to 2-fold. Behavioural support is usually provided without pharmacotherapy, however, because of concerns that drug therapy may harm the fetus [8]. This is understandable for bupropion, but is far less logical for nicotine.

Pregnant women who smoke will already expose their unborn children to nicotine. Nicotine has well documented potential adverse effects in pregnancy, since it is a vasoconstrictor and nicotine from cigarettes causes dose-related increases in maternal blood pressure and heart rate and has lesser effects on the fetal heart rate [9]. In rats chronic nicotine exposure is associated with dose-dependant alterations in behavioural and cognitive responses, CNS toxicity and a diminished adrenal response to hypoxia that, in humans, could pre-dispose to sudden infant death syndrome [9]. Consequently, nicotine may also be responsible for the attention deficit and learning problems that are described above [4]. Cigarette smoke, however, contains numerous other toxins in addition to nicotine and it is not known which of these actually cause harm, though the fetal effects of nicotine have been most widely studied. The cardiovascular effects of nicotine from NRT are less than those observed from smoking and regular NRT use generates lower plasma nicotine concentrations (when body weight is accounted for) than those in the animal experiments described above [9]. There is also no evidence that NRT use in pregnancy results in higher plasma nicotine concentrations than smoking [9]. For these reasons, and because using NRT in pregnancy results in exposure to only nicotine and no other toxins, there is expert consensus that NRT use is safer than smoking in pregnancy as long as pregnant women using NRT do not receive more nicotine from NRT than they would have done by smoking [10,11]. It is difficult, though, for health professionals to give clear guidance to pregnant women on using NRT when the safety of NRT in pregnancy is justified primarily on theoretical grounds and its efficacy has not been established.

To date, evidence on the effectiveness of NRT in pregnancy comes from 3 studies and is inconclusive [12-14]. Two of these studies were trials investigating NRT as transdermal patches. [12,13]. but one [13] was stopped after only 40 patients had been randomised. The other [12], however, randomised 250 women but produced no clear evidence that NRT was effective, since the odds ratio for smoking cessation using NRT versus placebo was 1.1 with a 95% CI of 0.7 to 1.8. This odds ratio is much lower than that obtained from meta-analysis of trials of NRT patches in non-pregnant subjects (OR, 1.74) [5] and raises questions about whether using NRT in pregnancy is effective for smoking cessation. The third study was not placebo controlled and randomised women to intensive behavioural support with an additional option to use NRT patches and/or gum).)[14] or a 'normal care' group which received only very minimal smoking cessation advice. Although, 75 women in this trial opted to use NRT, this design makes it difficult to disentangle any effect of NRT from that of intensive behavioural support. Where reported, no harmful effects of NRT were demonstrated in these 3 studies. In the larger patch trial [12], babies born in the NRT group were significantly heavier than others [mean birth weight (adjusted for prematurity) difference = 186 g (95%CI 35,336 g)], suggesting that pure nicotine as NRT has less impact on fetal growth in utero than smoking. Additionally, in the trial which allowed a group of women to use either NRT patches or gum or a combination of these, mean birth weights in fetuses born after 37 weeks were not statistically different between the 2 trial groups [non-significantly lighter (by 32 g) in NRT group]. In both trials that reported the distribution of low birth weight infants between groups [12,14], no significant differences were noted.

It has recently become apparent that conventional doses of nicotine contained in NRT may be insufficient for pregnant women and this may explain the negative findings from the one trial of NRT in pregnancy. In pregnancy, the metabolic clearances of nicotine and cotinine (the principal metabolite of nicotine) are increased by 60% and 140% respectively. [15]. Accordingly, even when pregnant women take standard doses of NRT for adequate periods, these may still be ineffective because they may require higher doses of NRT to replace the nicotine they would have received via smoking. Higher doses of NRT might, therefore, be needed in pregnancy, but because there is very little human-subject research into the effects of nicotine on the developing fetus, it is not known whether these might increase the risk of fetal damage. Until the effectiveness of the current conventional dose of NRT is established, it is hard to justify trials of higher ones.

In summary, although consensus opinion suggests that taking NRT during pregnancy is likely to be safer than smoking [8,10,11,16], there is little direct trial evidence to support this and we do not know if NRT is actually *effective *in promoting smoking cessation amongst pregnant smokers. The *SNAP *trial will produce direct evidence on these important questions and will investigate whether or not NRT is more effective than placebo in achieving smoking cessation for women who and are between 12 and 24 weeks pregnant, who currently smoke 5 or more cigarettes daily and who smoked 10 or more cigarettes daily before pregnancy.

## Methods/Design

Ethical approval to conduct this study from the Oxfordshire REC A ethics committee (ID number 04/Q1604/85)

### Treatment group

Pregnant women will receive an eight week course of 15 mg/16 hr NRT transdermal patches. Although many studies have used longer courses, there is no evidence that these are more effective [5]. Patches will be issued in conjunction with individual behavioural support (*Section 10*) which is an effective smoking cessation intervention in pregnancy [7]. Four weeks after their quit dates, women who are not smoking will be issued with a second four week supply of patches.

### Control group

Women in the control arm of the trial will receive an identical placebo NRT patch and the same behavioural support as those in the treatment group. In both control and intervention groups, participants will be blind to their group allocation.

### Randomised procedure

After collecting pre-randomisation baseline data, exhaled carbon monoxide readings will be taken from women and assuming that readings indicate that women do smoke [cut off 8 ppm [17]], informed consent for trial entry will be sought. After consenting to trial entry, women will receive an initial behavioural support session before being randomised. Full details are given later.

Randomisation will be via the Nottingham Trials Unit web-based database and randomisation service. In each centre the recruiting research midwife (RM) will have a username and password. (S)he will log on to the trial website that hosts the trial database, confirm that the patient eligibility criteria are all met and enter an agreed minimum amount of *registration *data about the participant and centre before randomisation is possible. Data to be entered at this stage are described later. The computer will then issue a trial number which will be the unique identifier for the trial participant and a trial pack number which will reflect the treatment allocated. Randomisation will be stratified by trial centre only.

Numbered packs of active and placebo patches will be distributed by Queens Medical Centre pharmacy and stored in all participating ante-natal/ultrasound clinics. After randomisation, the research midwife will select the patch pack with the appropriate number and issue this to the participant. The research midwife and the trial participant will both be blind to group allocation. The research midwife will issue NRT/placebo under the supervision of a senior, local doctor via a patient group directive (PGD) [18]. When research midwives visit women at home to enrol them into the trial, immediate internet randomisation will not be possible. In this circumstance the research midwife will return to her/his hospital base to randomise the enrolled woman and the appropriate trial pack will be posted to the trial participant.

### Outcome measures

These relate to smoking, fetal loss, fetal and maternal morbidity and health economic outcomes.

Primary end point: Self-reported, prolonged and total abstinence [19] from smoking or the use of any non-pharmacological nicotine containing substances between a quit date set within two weeks of randomisation and immediately prior to childbirth.

Prolonged abstinence cannot be comprehensively validated, but if participants report prolonged abstinence and are abstinent at ***both ***time points below, they will be considered tohave a positive primary outcome. This is **one **outcome requiring **three **different measures to be achieved.

i) Self reported smoking cessation for at least 24 hours before follow up at one month after quit date, ***validated by ***exhaled CO measurement [cut off 8 ppm [17]].

ii) Self reported smoking cessation for at least 24 hours before hospital admission for childbirth, ***validated by ***exhaled CO measurement [cut off 8 ppm [17]].

Secondary end points:

#### a) Smoking

1. Self reported, prolonged abstinence [19] from smoking between quit date and one month.

2. Self reported, prolonged abstinence [19] from smoking between quit date and 6 months after delivery.

3. Self reported smoking cessation for previous 7 day period at 6 months after delivery (point prevalence) [19].

4. Self reported, prolonged abstinence [19] from smoking between quit date and 2 years after delivery.

5. Self reported smoking cessation for previous 7 day period at 2 years after delivery (point prevalence) [19].

#### b) Fetal loss and morbidity

1. Fetal death and stillbirth

2. Neonatal death (i.e. from birth to 28 days)

3. Post-neonatal death (29 days to 2 years)

4. Individualized birth weight Z score (i.e. birth weight adjust for gestational age, maternal height, maternal weight at booking and ethnic group).

5. Apgar score

6. Cord blood ph

7. Gestational age at birth

8. Intraventricular haemorrhage

9. Neonatal enterocolitis

10. Neonatal convulsions

11. Congenital abnormality

#### c) Maternal morbidity and mortality

1. Maternal mortality

2. Mode of delivery

3. Proteinuria

4. Hypertension in pregnancy

#### d) Early childhood outcomes

1. Behaviour and development at 2 years

2. Disability at 2 years

3. Respiratory symptoms at 2 years

#### e) Health economic data

1. Duration of maternal hospital admission for childbirth

2. Duration of any admission (of baby) to special care

3. Health status at 6 months (EQ5D) [20]

### Sample size

We need to recruit 525 women into each arm of the study. A trial with 500 women in each arm would detect an absolute difference of 9% in smoking cessation rates between the two groups immediately before childbirth with a two-sided significance level of 5% and a power of 93%. We anticipate that up to 5% of women will be lost to follow up and inflate our sample size (of 500) by a factor of 1.05 to allow for this. This size of study would allow us to detect smaller treatment effects with lower power. For example, we would have 80% power to detect an absolute difference in cessation rates of 7%.

A Cochrane review has shown that approximately 10% of women who are still smoking at the time of their first antenatal visit will stop smoking with usual care and a further 6% to 7% will stop as a result of a formal smoking cessation program using intensive behavioural counselling [15]. This means that in our control group (*placebo plus intensive behavioural counselling*) we can expect a smoking cessation rate of around 16%. The most recent Cochrane review of NRT, reports a treatment effect (odds ratio) for transdermal patches of 1.74 95%CI (1.57–1.93) [5]. Consequently, if we were to find NRT as effective in pregnancy as it is generally, we could expect a smoking cessation rate of approximately 25% in our treatment group (*NRT plus intensive behavioural counselling*).

### Inclusion criteria

Eligible women are women between 12 and 24 weeks pregnant, who report smoking at least ten cigarettes daily *before *pregnancy and who *still currently *smoke at least five cigarettes daily. They also must have an exhaled CO reading at least 8 ppm. Women may only enrol into the trial once and may participate in other non-conflicting research projects.

### Exclusion criteria

Women with the following contraindications to the use of NRT will be excluded: severe cardiovascular disease, unstable angina, cardiac arrhythmias, recent cerebrovascular accident or TIA, chronic generalized skin disorders or known sensitivity to nicotine patches, chemical dependence/alcohol addiction problems. Also, women who cannot give informed consent and those with known major fetal anomalies will be excluded. IUGR is not an exclusion criterion.

### Recruitment (see Figure 1)

**Figure 1:**
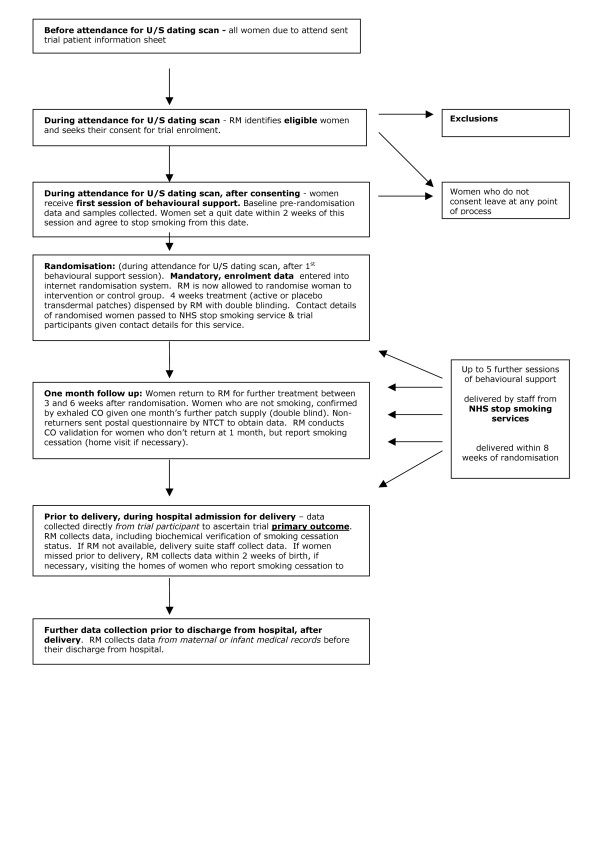
**Flow of trial participants from recruitment to delivery**. Research midwives in each centre are responsible for accurate data entry to internet hosted database, for sending blood samples to appropriate university departments and accurate paper copies of data collection sheets from i) baseline (pre-randomisation) ii) one month follow up and iii) delivery to the Nottingham Trial Co-ordinating Team.

All pregnant women between 12 and 24 weeks into pregnancy who smoke and are interested in stopping smoking are potentially recruits to the study. Brief information about the trial and patient information sheets (PIS) will, therefore, be posted to all women who attend trial site hospitals for ante natal care with their routine antenatal ultrasound scan appointment letters (scans are usually performed at between 12 and 20 weeks gestation). In each trial hospital, a w.t.e. research midwife (RM) working with non-w.t.e. clerical assistant will use a systematic method to identify smokers who are interested in participating from all women attending for ultrasound examinations. During piloting a questionnaire was used for this and a similar instrument could be used in any or all of the 5 trial centres. The final method of identifying eligible patients will be agreed with the Chief Investigator. Research midwives will also agree a method for monitoring the numbers of women identified as potentially eligible to join the trial and the proportion of these that eventually enrol.

### Consent

Women who are interested in participation will be asked to discuss this with the research midwife. The research midwife will ascertain if women are eligible to join the study and have read the PIS at least 24 hours earlier. If they have read the PIS, the research midwife will answer any questions that women have about trial enrolment and seek informed consent to:

i) trial participation

ii) collection of follow up data on materno-fetal outcomes from medical records

iii) participants' registration with the Office for National Statistics

iv) collection of a blood sample for cotinine estimation and DNA extraction & storage

v) collection of saliva samples for cotinine estimation

vi) potential future contact for follow up studies by University of Nottingham based investigators

If women have not read the PIS, but express an interest in the study, they will be given a copy and contacted after 24 hrs to determine whether or not they consent to enter the trial. These women will be contacted after 24 hrs and if they are still interested in enrolling in the study, informed consent will be sought.

Once consent is recorded, baseline data, saliva, blood samples and exhaled CO readings are obtained. Next the research midwife delivers the first session of behavioural support to the participant during which a quit date which is within 2 weeks when they will start using transdermal patches is agreed.

### Registration & randomisation

Immediately after the behavioural support session, the research midwife uses a PIN to log on to the University of Nottingham internet randomisation service and enters the ***mandatory enrolment data ***below, without which randomisation will not be permitted. The participant is automatically allocated a trial number (i.e. unique ID) and a trial treatment pack number which identifies the treatment required and the RM issues the corresponding trial treatment pack.

It is anticipated that around 50% of trial participants will need a home visit for intensive behavioral counseling and subsequent randomization. In this situation, the research midwife (RM) will ensure that all base line data including the ***mandatory enrolment data ***is collected whilst visiting the participant. The RM will return to base and randomization the participant via the internet before posting an appropriate treatment pack to the study participant.

The research midwife then sends letters to the participant's general practitioner and hospital obstetrician to inform them that she is enrolled in the trial. One copy of the consent form is placed in the hospital medical records, another accompanies the letter to the GP and the third is sent to the Trial Office.

### Further behavioural support

The research midwife will give the participant contact details for the local NHS stop smoking service and also pass the participants' details to this service. Participants will receive up to 5 further behavioural support sessions from the NHS stop smoking service according to an agreed format.

### Data handling

RMs will enter the data which they collect on to a secure database hosted by the University of Nottingham via an internet connection and will also make paper copies of data collection to allow audit. Once data collection at any one time point (e.g. baseline or one month) is complete, the research midwife will photocopy the data collection sheet and post a copy to the Trial Office. Infant records within the database will be created from within maternal ones and will automatically be linked to maternal and sibling trial records.

### Biological samples

i) For DNA extraction, 2 × 5 ml EDTA blood samples are required. These can be refrigerated or frozen (if later than 24 hrs elapses between collection and dispatch). If frozen, this needs to be to -20° centigrade. Samples will be dispatched to Professor Ian Hall at the University of Nottingham for long term archiving. Frozen samples require non-glass tubes.

ii) Blood for serum cotinine estimation (5 ml sample minimum) need to be placed in BD Gold top tubes (or equivalent). These need to be frozen as per i) above before transport to the Nottingham Trial Coordination Team prior to dispatch to Professor Michael McCoughtrie at the University of Dundee.

iii) Saliva for salivary cotinine estimation is also transported to Dundee after collection.

All frozen samples need to be transported on ice in non-glass containers labelled with:

• Trial number

• Hospital number

• Subject's initials

### Withdrawal from patch treatment

If for any reason, a participant terminates patch treatment, every effort must still be made to collect follow up data.

### Follow-up at one month after agreed quit date

Participants will return to the research midwife (RM) for further supplies of patches. To allow some flexibility this follow up will occur between 3 and 6 weeks after randomisation. The RM will ascertain women's smoking status and those who report not smoking regularly (confirmed by exhaled CO measurement) will be issued with a new trial treatment pack number (obtained by the RM from the online database) and will receive a corresponding treatment pack (containing 4 weeks' patches). A saliva sample to measure cotinine levels on treatment will be taken. The Trial Office will send a postal questionnaire asking about smoking status to women who do not return at one month. One postal and telephone reminder will be used. When women report continued smoking cessation and do not attend for further NRT, the research midwife will contact them to arrange CO validation of this, visiting them at home, if necessary.

### Follow-up immediately before childbirth

When participants are admitted to hospital whilst in established labour *prior to childbirth*, Delivery Suite staff will contact the research midwife who will visit participants to ascertain their self-reported smoking status and use of transdermal patches. Women who report abstinence from smoking in the previous 24 hours will be asked by the research midwife to perform exhaled CO testing and provide a saliva sample for cotinine estimation. The RM will have overall responsibility for data collection and will arrange with Delivery Suite staff for this to be obtained in her/his absence. The RM will telephone those missed whilst in hospital as soon as possible afterwards (within 2 weeks maximum) to collect smoking behaviour data. Where participants report smoking cessation, the research midwife will measure their exhaled CO readings and obtain a saliva sample for cotinine estimation, visiting women at home if necessary.

Further infant, fetal and maternal data will be obtained from medical records (*details below*)

### Data monitoring by RM between data collection points

These data are required to ensure that the Data Monitoring and Ethics Committee is provided with adequate information to form an opinion concerning trial safety:

Development of major fetal abnormality between randomisation and labour onset

Fetal death between randomisation and labour onset

Maternal death between randomisation and labour onset

Hospital admission

Each month the Trial Office will provide RMs in the 5 centres with a list of trial numbers for participants who are still pregnant. The RM will use these to access subjects' computer records to obtain the information listed above. In the event of a hospital admission the RM will assess whether or not a serious adverse event has occurred and act accordingly see below. If the RM enters a fetal death into the database, this will automatically prevent further infant follow up and the RM will liase with the mothers' obstetrician to determine whether or not asking for follow up information concerning smoking behaviour around the anticipated time of delivery is acceptable. Major fetal abnormalities will also be reported to the trial office who will review these individually before deciding whether or not the participant should be allowed to continue within the trial.

### Registration with Office for National Statistics

The Trial Office will "flag" participants (women and babies) with the Office for National Statistics (ONS) at birth to facilitate follow up. Each week during the 2 year follow up period, the ONS will inform the trial team of any post-neonatal (i.e. between 29 days and 2 years) or maternal deaths.

### Procedure for administering postal follow up questionnaires

**Figure 2:**
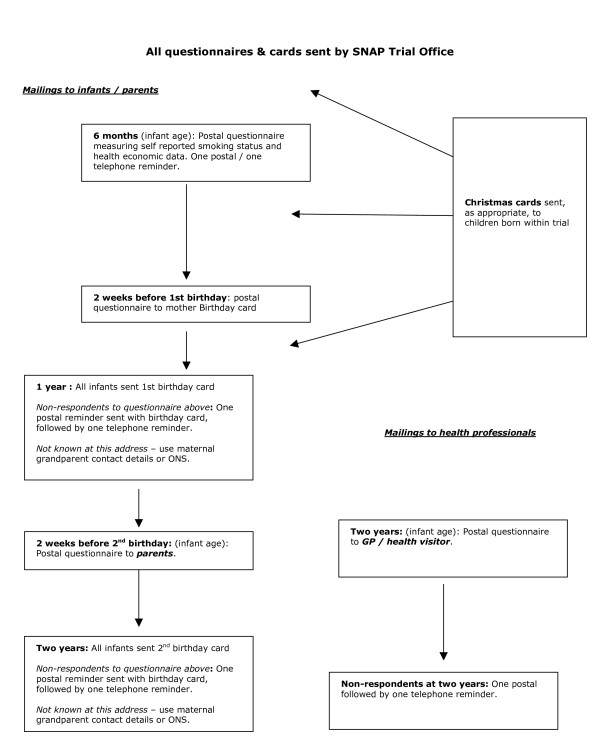
Follow up of trial participants from delivery until infants' second birthdays.

(see Figure 2): After infant deaths, questionnaires will not be sent and, where maternal deaths are reported, infants' general practitioners will be consulted about the appropriateness of continued follow up. The Trial Office will send questionnaires directly to study participants using contact details provided at study recruitment. For non-respondents or where questionnaires are returned labelled "*not at this address*", the office will check participants' addresses by contacting infants' grandparents and, if necessary, the ONS. ONS will trace the infant or mother and provide details of the Primary Care Trust (PCT) which provides their NHS health services and the Trial Office will then contact the infant's general practitioner so that a questionnaire can be sent. To maintain contact between researchers and participants, study infants will be sent Christmas cards and first birthday cards.

### Follow-up 6 months after childbirth

A postal questionnaire, with one postal and one telephone reminder, will be used to collect the data items specified below.

### Follow-up 1 year after childbirth

Two weeks before infants' 1st birthdays, *parents *will be sent a questionnaire to collect the data items specified below. Two weeks later, all participating infants will be sent a 1^st ^birthday card with a questionnaire reminder for non-respondents.

### Follow-up 2 years after childbirth

i) *Parent questionnaire*: Two weeks before infants' 2^nd ^birthdays, we will dispatch to *parents *a questionnaire to measure child behaviour, development, hospital admissions, respiratory symptoms and maternal smoking behaviour. Two weeks later, all participating infants will be sent a 2^nd ^birthday card with a questionnaire reminder for non-respondents. The instrument uses standard questions to record parents' reports of infants' respiratory symptoms [21] and behaviour [22], developed from those used in the MRC-funded UK Oscillation Study [23], with reference to the evidence base for questionnaire design [24].

ii) *Health professional questionnaire*: At the same time as parents' questionnaires are dispatched, we will post participants' general practitioners (GPs) a health professional questionnaire (*HPQ*) to measure children's disability according to a standard definition. [25] This brief instrument is designed to be completed using medical records. Health professionals completing these questionnaires require relatively little knowledge of the patient and GPs will be asked to complete them. If GPs cannot complete HPQs, they will be asked to forward these to children's' health visitors (HV). We will use an initial postal and subsequent telephone reminder to GPs to obtain the required information. The HPQ questionnaire has been validated and used with GPs and HVs previously [26,27] and requests data that HVs collect during their routine clinical practice to provide external validation of parents'.

### Data collection

Here the specific items of data to be collected at different points during the trial are outlined.

#### a) Baseline (i.e. pre-randomisation) data collection

Although online forms will allow data to be inputted to an online database, a paper copy of data will be kept for audit purposes.

##### i) Mandatory enrolment data (i.e. required for randomisation)

The RM will collect the following data from participants immediately after obtaining informed consent. The RM **must **enter the following data items about participants to the online database **before **randomisation is permitted:

*DoB *(valid range equiv to age 14–50)

participant's initials

hospital number

*daily number of cigarettes smoked before pregnancy*[28]

*daily number of cigarettes smoked currently*[28]

*agreed gestational age at time of randomisation *(valid range 12^0^–24^6^) [estimated delivery date will be calculated automatically within database]

time elapsed since last cigarette

exhaled CO reading of at last 8 ppm

*blood sample requested *(for cotinine assay, DNA extraction & storage)

indication that patient has signed consent form

*indication that participant's contact details have been recorded on paper *(see below)

agreed quit date

##### ii) Remaining baseline data for online entry

The following data will be collected with above data and the RM will also enter this on to the online database but entering these variables will **not **be mandatory before online randomisation is permitted.

*NHS number *(for ONS registration)

ethnic group

age left full time education

*number of previous births beyond 24 weeks *(valid range 0–12)

*time to first cigarette of day *[29]

partner's smoking status

maternal height

maternal weight at booking appointment

saliva sample

##### iii) Baseline data stored on paper

These personal data will be recorded on paper to allow data protection as the internet is not a secure transport medium.

*Participant name and contact details *(including landline/mobile telephone number & postcode)

*previous surname(s) *– for ONS registration

*Participant's general practitioner and/or name of practice plus practice address grandparents' contact details*, including phone numbers

#### b) One month after quit date

RM collects data from those who return. Postal questionnaires sent from NTCT to those who do not. The following data are collected:

RM notes *whether or not follow up occurs *and the *date of any follow up*. RM also inspects participants' supply of patches to calculate the number used.

Smoked at all in the previous 24 hrs

*Smoked since quit date *(further details on outcomes form 1)

Exhaled CO reading

On how many days have patches been used?

On how many days (if any) have non-trial patches been used?

How many behavioural support sessions with NHS stop smoking services used (telephone & face to face)?

Saliva sample for cotinine estimation taken

#### c) Upon admission for childbirth

The following data are recorded by the RM or delivery suite staff:

Date of follow up/exhaled CO reading or saliva sample

Smoked at all in the 24 hrs prior to delivery

Smoked between quit date and delivery

One or both of, *i) exhaled CO reading & ascertainment date ii) saliva sample *(for cotinine)

On how many days have patches been used?

On how many days (if any) have non-trial patches been used?

How many behavioural support sessions with NHS stop smoking services used (telephone & face to face)?

i) These data obtained by RM from ***maternal ***or ***infant medical records before ***discharge. For reasons of data protection some personal infant details are recorded on paper rather than being transmitted over the internet to the trial database:

*maternal *

hypertension (>140/90) on 2 occasions (excluding labour)

miscarriage (between randomisation and 24 weeks)

labour onset (spontaneous, induced, no labour)

mode of delivery (SVD, instrumental, caesarean)

ante natal or post natal maternal hospital admission

*infant*

baby initials

D.O.B

Gender

Baby NHS number

*Prompt for RM to confirm full name and address and contact details of baby and to record these on paper *(see below)

Prompt for RM to make a new record of contact details if these have differed from previous (i.e. maternal) ones

baby hospital number

birth weight

Number of births

if multiple birth, indicate number and birth order

live or stillbirth?

cord ph < 7.0

Apgar <7 at 5 min

*Gestational age at birth *– to be calculated within database from gestation at recruitment

These infant personal details will be recorded on paper only:

baby name

baby address (inc postcode)

ii) These data obtained by research midwife from ***infant medical records after ***discharge:

If live birth? live on leaving hospital

ventilation > 24 hrs

necrotising enterocolitis

neonatal convulsions

admitted to special care

*intraventricular haemorrhage *(4 categories)

*congenital abnormality present (y/n). If y then free text to describe this*.

#### d) Six months after delivery

The following data will be requested by postal questionnaire, dispatched from the trial office:

Smoking status

Length of maternal inpatient stay for delivery of > 24 hours duration (if any)

Any infant neonatal admission to special care

Length of any infant inpatient stay on special care

*Maternal use of NRT/NHS stop smoking services since childbirth*,

*EQ5D questionnaire *[20]

#### e) At 1 year after delivery

The following data will be requested: *smoking status, respiratory symptoms and infant hospital admissions for respiratory illness and other causes*

#### e) At 2 years after delivery

The following data will be requested:

***Parent questionnaire**** – Smoking status, infant behaviour, development, respiratory symptoms and hospital admissions*.

***Health professional questionnaire**** – Child's disability*

### Interventions

Details of NRT patches are given earlier in this document and details of behavioural support follow. The first behavioural support session will be provided at recruitment by a research midwife who has been trained in smoking cessation methods in accordance with national standards [30] and who has dedicated time for this task. Models of behavioural support that are effective in pregnancy vary greatly[7] and in non-pregnant subjects, behavioural support following very different psychological models are all equally effective [31]. We will, therefore, standardise the first support session to include information on:

i) the harmful effects of smoking in pregnancy

ii) the role of nicotine addiction in sustaining smoking

iii) how to use NRT (including safety concerns)

iv) coping with withdrawal symptoms.

Support will be specific to the needs of pregnant women and may involve:

i) enlisting partner support

ii) a partner quit attempt

iii) ensuring that the partner has information about smoking cessation services

Study midwives will use brief cognitive – behavioural counselling, combining components from effective counselling strategies that are effective [31], such as:

i) providing structure to quit attempts

ii) agreeing a "contract" for any attempt

Counselling will be delivered in a manner that is consistent with routine clinical practice, using a format similar to that which has been effectively applied within routine ante natal care in the US [32]. A quit date which is within 2 weeks will be agreed and participants will be instructed to start using patches on this date.

Local NHS stop smoking services will provide up to 5 *subsequent behavioural support sessions*. These follow up sessions will reinforce women's reasons for quitting and strategies for success. A standardised approach to follow up support sessions is important and NHS stop smoking service staff will be orientated towards this.

### General statistical analysis plan

#### a) Primary outcome measure

The proportion of women who report prolonged and total abstinence from smoking immediately before child birth will be compared between treatment groups by Chi-squared test, on an intention to treat basis (all those randomised) with smokers lost to follow up considered to have continued smoking. For this analysis, we will assume that women in each group use their allocated treatments as directed and no randomised participants will be excluded from analyses. Baseline data on smoking behaviour and demographic information will be compared between groups, and adjustment made for any differences, using logistic regression.

#### b) Child behaviour and development scores at 2 years

We will compare in children born to women in the control and intervention groups, using t-test (via log transformation) or the Mann-Whitney U statistic. Again this will be done on an intention to treat basis. A small number of children will be born as multiple births (e.g. twins) and data for these cases will be clustered rather than independent. Robust standard errors, or a similar appropriate statistical method will be used in analysis of child data to allow for this.

There will be two analyses. The first will be conducted upon data obtained around delivery. The second will be conducted at 2 years after delivery, using data obtained between delivery and this time point. Data collected for secondary outcomes will not be analysed until the trial has ended with respect to the primary outcome measure.

#### c) Other outcomes

i) *Fetal birth outcomes *and ii) *Maternal birth outcomes *will also be compared on an intention to treat basis between the 2 groups in the first analysis at delivery (as *a *&*b *above)

As these outcomes relate to the safety of NRT in pregnancy we will also conduct an analysis of these outcomes comparing participants in each group who report using any patches with those in each group who report using none.

#### d) Sub group analyses

These will be conducted to investigate the relationship between i) baseline cotinine levels and cessation and ii) maternal educational level (proxy for socio-economic status) and cessation. We will model the relationship between smoking cessation, pre-treatment plasma cotinine levels and treatment group in a logistic regression, to establish whether there is effect modification by pre-treatment plasma cotinine and whether efficacy at given levels of plasma cotinine varies. The model will also establish whether or not smoking cessation is constant across all levels of pre-treatment plasma cotinine in the NRT group, or reduces with increasing pre-treatment plasma cotinine, which could be indicative of inadequate replacement of nicotine. We will use similar methods to investigate ii) above.

### Health economic analysis plan

Economic analysis will be undertaken to investigate short term and longer term potential cost-effectiveness of NRT in pregnancy. The cost-effectiveness of NRT use by the general population has been established [33] and a small number of studies have investigated the potential cost saving of smoking cessation interventions in pregnancy. [34], but few have used empirical data on costs of interventions. Analyses for this study will be primarily undertaken from an NHS perspective. Uptake of behavioural support and NRT will be monitored and costs of both estimated with both locally-specific and national average values. The differential consequences in terms of length of maternal stay and post natal delivery to special care between the two arms of the trial will be used with the estimated costs of delivering interventions with and without NRT patches and differential smoking cessation rates to estimate the incremental cost-effectiveness ratio. Sensitivity analyses exploring assumptions made in estimating the control state (no NRT) will be undertaken. The primary health outcome will be maternal smoking cessation immediately before delivery and differences in health status at 6 months (from EQ5D data) will be converted into QALYs to allow cost-utility modelling. Additionally, a range of modelling techniques will be used to estimate longer-term cost-utility from two year follow-up data. Epidemiological and economic models will be used to estimate lifetime gains in QALYs from smoking cessation and savings in health care expenditures [33,35]. A full literature review will be undertaken to explore the potential for providing monetary estimates of the long term impacts on the child of their differential birth outcomes.

### Safety

To minimise the likelihood of women or infants being harmed by unexpected effect(s) of nicotine that could not predicted from previous research, the Data Monitoring & Ethics Committee will have access to birth outcome data. These data will be available for the DMEC to analyse as is considered appropriate to investigate whether or not significant or clinically-important differences arise between study groups (e.g. in birth weight).

### Safety reporting

The following will be considered ***adverse events *(AEs)**:

Withdrawal from patch treatment due to i) skin reaction or ii) other symptom(s) which are potentially caused by NRT (listed in section 4.10 BNF)

AEs will be reported in an annual safety report to the MHRA, REC and Sponsor.

The following will be considered ***Suspected Unexpected Severe Adverse Reactions ***(SUSARs):

*Baby*: fetal death, still birth, neonatal and post-neonatal death, congenital abnormality, special care admission (excluding transitional care admission)*

*Maternal*: eclampsia, maternal death, some hospital admissions* (see below)

*Birth outcome*: placental abruption, premature birth (earlier than 32 weeks)*, low birth weight (< 2,500 g)

The following hospital admissions are **not** SUSARS: admission for delivery, uncomplicated false labour, unconfirmed fetal compromise (i.e. baby well), vaginal bleeding of no serious cause and infant hospital admissions ***after*** the neonatal period

Any other serious unexpected event.

All SUSARs except those marked with an asterisk* are considered life threatening.

Life threatening or fatal SUSARs (no asterisk) will be reported to the MHRA and REC within 7 days (follow up report within 15 days) and also to relevant NHS trust R&D office according to local policies.

Non life threatening SUSARs (asterisk) will be reported to the MHRA and REC within 15 days and also to R&D offices, as appropriate.

SUSARs will also be reported to the DMEC chair along with the treatment allocation group of the trial subject and a cumulative count of SAE and SUSAR frequency in each trial arm.

#### Trial steering committee

**Table 1:** 

Mr Peter Brocklehurst (Chair)	Director, National Perinatal Epidemiology Unit, University of Oxford
Professor Peter Hajek	Professor of Clinical Psychology, Tobacco Dependence Research Centre, Barts and The London, Queen Mary's School of Medicine and Dentistry
Dr Carol Coupland	Senior Lecturer in Medical Statistics, Division of Primary Care, University of Nottingham
Mrs Sue Maguire	Lay member
Dr Michael Murphy	Director, Childhood Cancer Research Group, University of Oxford

#### Data monitoring and ethics committee

**Table 2:** 

Professor Janet Peacock (Chair)	Professor of Health Statistics, Brunel University
Professor Khalid Khan	Professor of Obstetrics, Gynaecology and Clinical Epidemiology, University of Birmingham
Professor David Field	Professor of Neonatal Medicine, University of Leicester

### Funding

The research costs of SNAP are funded by the NHS Health Technology Assessment Programme and the NHS Support Costs have been met from NHS R&D funds.

## Discussion

Recruitment to the SNAP trial will start in 2007 and at the conclusion of the trial, it should produce valuable information about the safety and efficacy of NRT use in pregnancy.

## Competing interests

All authors apart from John Britton (JB) and Tim Coleman (TC) have no competing interests. In the last 5 years, JB has been paid by the following drug companies that manufacture NRT. Pharmacia paid for consultancy work (on one occasion). Pfizer paid funds to the University of Nottingham (i.e. not for personal gain) for work as a PI in a multi-centre study. Pfizer also paid University of Nottingham for JB's time to give talks at fringe meetings of political parties conferences' (Labour and Conservative) in 2004. GlaxoSmithKline (GSK), an honorarium was paid via a third party organisation for talking at a conference.

Over 5 years ago TC was paid by Pharmacia for one piece of consultancy and GSK for talking at a conference on smoking cessation.

## Authors' contributions

TC, JT, JB, SL, KW, MMcC, CM, NM and CG all helped develop this research protocol, contributing expertise within their own particular knowledge base. Contributions were in the following fields: smoking cessation (TC, JB, SL, CM, KW), clinical trials (JT, JB, SL, TC, CM, NM, CG and CM), midwifery and obstetrics (KW and JT), neonatology/paediatrics (NM), statistics (SL), health economics (CG) and biological measurement of cotinine (MMcC). All authors have read and approved the final manuscript.

## Pre-publication history

The pre-publication history for this paper can be accessed here:


